# Automation bias in electronic prescribing

**DOI:** 10.1186/s12911-017-0425-5

**Published:** 2017-03-16

**Authors:** David Lyell, Farah Magrabi, Magdalena Z. Raban, L.G. Pont, Melissa T. Baysari, Richard O. Day, Enrico Coiera

**Affiliations:** 1 Centre for Health Informatics, Australian Institute of Health Innovation, Macquarie University, Sydney, NSW 2109 Australia; 2 Centre for Health Systems and Safety Research, Australian Institute of Health Innovation, Macquarie University, Sydney, NSW 2109 Australia; 3 St Vincent’s Clinical School, Faculty of Medicine, University of New South Wales, Sydney, Australia; 4 St Vincent’s Hospital Clinical School and Pharmacology, School of Medical Sciences, Faculty of Medicine, University of New South Wales, Sydney, Australia

**Keywords:** Decision support systems, Clinical, Cognitive biases, Complexity, Electronic prescribing, Medication errors, Automation bias, Human-computer interaction, Human-automation interaction

## Abstract

**Background:**

Clinical decision support (CDS) in e-prescribing can improve safety by alerting potential errors, but introduces new sources of risk. Automation bias (AB) occurs when users over-rely on CDS, reducing vigilance in information seeking and processing. Evidence of AB has been found in other clinical tasks, but has not yet been tested with e-prescribing. This study tests for the presence of AB in e-prescribing and the impact of task complexity and interruptions on AB.

**Methods:**

One hundred and twenty students in the final two years of a medical degree prescribed medicines for nine clinical scenarios using a simulated e-prescribing system. Quality of CDS (correct, incorrect and no CDS) and task complexity (low, low + interruption and high) were varied between conditions. Omission errors (failure to detect prescribing errors) and commission errors (acceptance of false positive alerts) were measured.

**Results:**

Compared to scenarios with no CDS, correct CDS reduced omission errors by 38.3% (*p* < .0001, *n* = 120), 46.6% (*p* < .0001, *n* = 70), and 39.2% (*p* < .0001, *n* = 120) for low, low + interrupt and high complexity scenarios respectively. Incorrect CDS increased omission errors by 33.3% (*p* < .0001, *n* = 120), 24.5% (*p* < .009, *n* = 82), and 26.7% (*p* < .0001, *n* = 120). Participants made commission errors, 65.8% (*p* < .0001, *n* = 120), 53.5% (*p* < .0001, *n* = 82), and 51.7% (*p* < .0001, *n* = 120). Task complexity and interruptions had no impact on AB.

**Conclusions:**

This study found evidence of AB omission and commission errors in e-prescribing. Verification of CDS alerts is key to avoiding AB errors. However, interventions focused on this have had limited success to date. Clinicians should remain vigilant to the risks of CDS failures and verify CDS.

**Electronic supplementary material:**

The online version of this article (doi:10.1186/s12911-017-0425-5) contains supplementary material, which is available to authorized users.

## Background

The electronic prescription of medicines (e-prescribing) is now routine, [1] making the clinical decision support (CDS) systems they include [2] amongst the most common encountered by clinicians. CDS can help reduce adverse events by displaying alerts for potential errors such as drug-drug interactions [3–5].

However, CDS is not perfectly accurate and will at times provide inaccurate advice [6]. Over-reliance on alerts may cause clinicians to avoid prescribing particular medicines due to inappropriate alerts or clinicians may fail to detect prescribing errors with the potential for harm because they were not alerted to them.

This over-reliance on CDS is referred to as automation bias (AB), and is defined as “the tendency to use automated cues (such as CDS alerts) as a heuristic replacement for vigilant information seeking and processing [7].” With AB omission errors, users fail to notice problems because they were not alerted to the problem by CDS, and with commission errors, users comply with incorrect recommendations [7]. There are multiple possible causes of AB, [8, 9] and the literature is currently unclear regarding which, or all, of these are genuinely causal, and under which circumstances. For example, commission errors have been associated with reduced sampling of information which can verify decision support [10, 11]. However, human factors studies have found that some individuals make commission errors *despite* sampling all required information [12, 13]. This has been described as a ‘looking but not seeing’ effect, suggesting that human information processing is also a factor affecting AB.

The majority of AB research comes from the human factors and ergonomics literature, mostly focused on aviation and process control [14]. There have been a small number of studies conducted in healthcare, finding evidence of AB omission errors in computer-aided detection of cancers in mammograms, [15, 16] and commission errors in the computerized interpretation of EKGs, [17] and answering questions about clinical scenarios [18]. Goddard, et al. [19] found evidence of commission errors, where general practitioners answered questions about which drugs they would prescribe for different clinical scenarios. They found a significant effect for participants changing from correct to incorrect responses after being provided with incorrect CDS advice.

For e-prescribing systems, decision support is commonly provided in the form of alerts that warn clinicians about potential prescribing errors [2]. Despite such alerts being one of the most common forms of decision support, the high volume of prescriptions ordered, and risk of harm to patients from prescribing errors, no studies have yet assessed the risk of AB in e-prescribing.

The prevailing view in the human factors literature is that AB only occurs in a multi-task environment [14, 20, 21]. However AB has been reported in some, but not all, tasks in a single task environment [14]. The discrepancy between single tasks which do and do not produce AB suggests that properties of the task itself may be risk factors for AB. The occurrence of AB may be related to how complex it is to verify that automation is working correctly, and that complexity across multiple simultaneous tasks appears to be cumulative [14]. In addition to multitasking, clinical settings are very prone to interruptions, requiring the clinician to switch between their primary task and the interruption, introducing increased cognitive workload and task complexity [22]. However, to date, no studies have tested the impact of interruptions on AB.

This study seeks to test for the presence of AB in e-prescribing assisted by CDS, which provides decision support in the form of alerts for prescribing errors. Additionally, it seeks to test the impact of interruptions and task complexity on AB. In doing so we seek to understand: (1) The baseline impact of correct CDS alerts on prescribing errors; (2) The impact of CDS false negatives on omission errors; (3) The impact of CDS false positive alerts on commission errors; (4) The impact of interruptions on AB; (5) The impact of task complexity on AB.

## Methods

### Participants

One hundred and twenty students enrolled in the final two years of a medical degree at Australian universities participated in the study. Australian medical education uses an integrative approach where students learn patient and clinical content throughout their degree. By the final two years of their education, participants would have typically received training in rational and safe prescribing. They also complete the National Prescribing Curriculum, a series of online modules based on the prescribing principles outlined in the World Health Organisation’s Guide to Good Prescribing [23]. Upon completion of these final two years, graduates would begin practice as junior medical officers.

Participants responded to advertisements emailed by medical schools or posted on social media via medical students’ societies. Ethical approval was granted by the ethics committees of Macquarie University and the University of New South Wales. Participants were offered two movie vouchers and a certificate for their participation.

### Experiment design

**Fig. 1:**
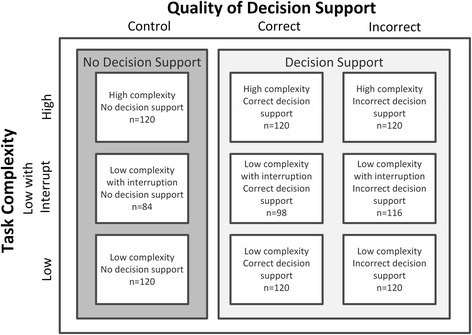
Experimental design with the number of participants in each condition. All participants completed all conditions. However, some were excluded from the analysis of interruption conditions as they did not trigger the interruption task

The study had two within-subject factors: quality of CDS (correct, incorrect and no CDS) and task complexity (low, low with interruption and high) providing nine conditions (Fig. 1). Each participant received all nine conditions, completing one scenario in each condition. The experimental control were scenarios presented to participants with no CDS.

The allocation of the nine prescribing scenarios to the nine experimental conditions, the order of presentation, and whether participants received control scenarios first or last were randomized. The position of prescribing and false positive errors in the list of medicines to be prescribed was randomised, allocated at the time of scenario design. The position of alerts was varied depending on the CDS condition that was randomly allocated to the scenario for each participant at the time of enrolment.

### Experimental task

**Fig. 2:**
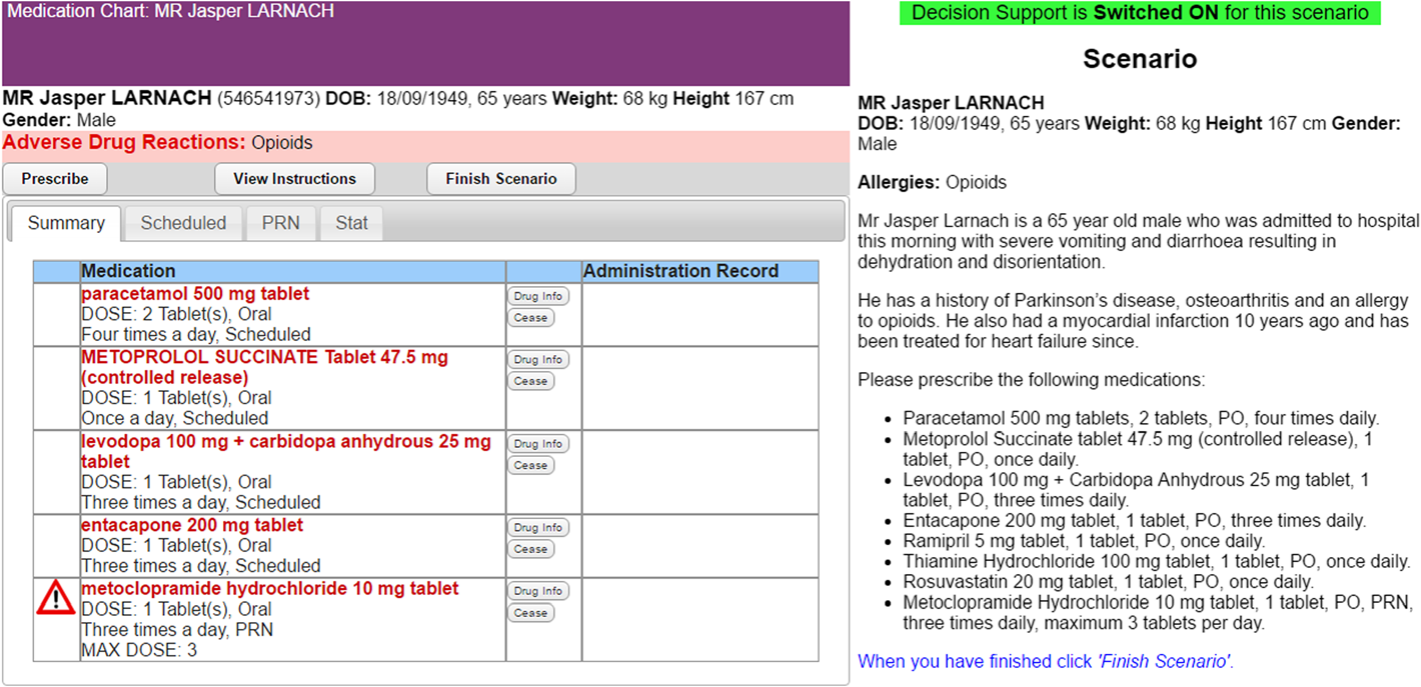
The e-prescribing system interface and scenario

Figure 2 provides an example of the participants’ task in this experiment. Participants were presented with nine prescribing scenarios for which they were asked to prescribe medicines using an e-prescribing system. Each scenario presented a brief patient history together with a list of medications to prescribe.

The prescribing scenarios were developed with advice from an expert panel, including four hospital doctors, a medical pharmacology registrar and two pharmacists (including MZR). They were independently reviewed by a consultant physician specialising in pharmacology (RD), to ensure clinical relevance. The scenarios presented hypothetical patient scenarios and involved prescribing tasks that were typical of those undertaken by junior medical officers, based on observations of e-prescribing in a medical ward of a major teaching hospital. A common task performed by junior medical officers is the prescribing of medications using an e-prescribing system upon admission of a patient to hospital, including medicines taken prior to, and those initiated on admission.

Each scenario included one genuine prescribing error, where one of the medicines was clinically contraindicated in that scenario (Additional file 1: Appendix A). These were designed to be unambiguously errors and of sufficient severity in the risk posed to the patient that the medicine should be avoided under all circumstances. To ensure this, the severity of the errors included in the scenarios were independently assessed by a clinical pharmacist (LGP). The error in one scenario was assessed as potentially lethal, five were serious, and three were significant [24]. All other medicines listed in scenarios where carefully chosen so as to be unambiguously free from error.

Scenario complexity was manipulated by varying the amount of information contained in the prescribing scenarios [25]. The nine scenarios were divided into six low-complexity scenarios (each containing six information elements) and three high complexity scenarios (each containing seventeen elements). An information element was classified as either a condition, symptom, test result, prior treatment, allergy, observation, or requested prescription. Each element could potentially interact with other elements in a way that could result in a prescribing error, for example, drug-drug interactions and conditions which may contraindicate the use of a particular medicine. The more elements, the more potential interactions the participant needs to assess. Low-complexity scenarios contained a list of three requested medicines, while high-complexity ones contained eight. The number of elements in each scenario was coded by DL and reviewed by MZR; disagreements were resolved by consensus.

In interruption conditions, participants were interrupted, once per scenario, whilst viewing drug information and presented with a task requiring a response before they could continue. The task required them to seek out and retain in memory three information elements to calculate a dose (Additional file 1: Appendix B).

### E-prescribing system

A simulated e-prescribing system was developed which allowed for the manipulation of the triggering and content of CDS alerts. This web-based system was presented to participants as being in development. A medication administration record was not implemented, nor were participants required to specify times of administration.

**Fig. 3:**
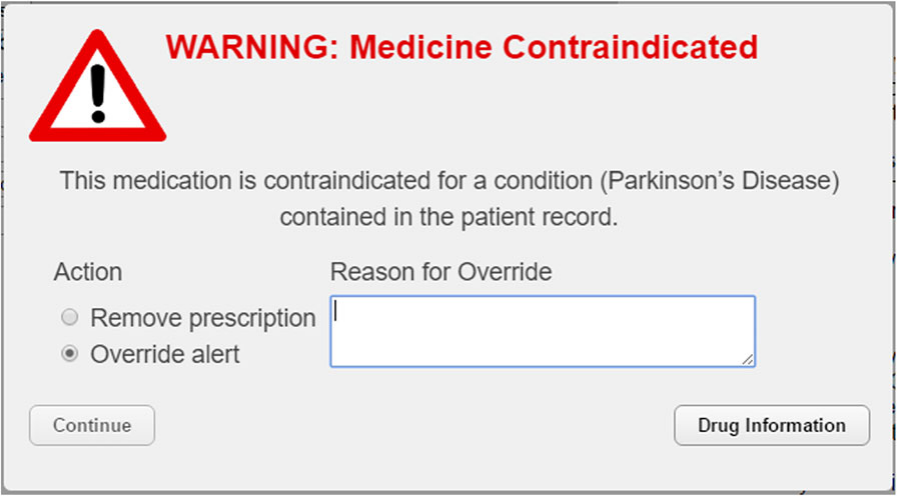
CDS Alert

CDS was provided in the form of alerts (Fig. 3) which were triggered once a prescription was entered. The alert provided a generic warning about the nature of the error, followed by specific details.

Participants could resolve the alert by choosing either to remove (i.e., not prescribe) the medicine or to override the alert with a reason and prescribe that medicine anyway. The alert also provided direct access to drug information for the relevant medicine from the Australian Medicines Handbook [26]. The Australian Medicines Handbook references the Australian formulary and is a gold standard medicines reference. It is evidence-based, reflects Australian best practice and is widely utilised in Australian clinical practice [27]. This reference was also readily accessible from the medication chart and in prescription order entry screens and could be used to identify prescribing errors and verify the information provided by CDS alerts.

The quality of CDS provided to participants was manipulated across conditions:

- *Correct CDS* alerts triggered only by genuine prescribing errors (true positives). Due to the severity of the prescribing errors, all correct alerts were highly relevant. The absence of alerts always indicated true negatives.
- *Incorrect CDS* failed to alert the genuine prescribing error (false negative) and provided one false positive alert, per scenario, for a medicine that was safe to prescribe.
- *No CDS* served as the control condition in which there was no CDS checking for errors. Participants were informed of this and advised to use the drug reference to identify errors.

### Procedure

After having given informed consent, participants completed a pre-experiment questionnaire and watched a brief instructional video on how to use the e-prescribing system. The video included a demonstration of the correct functioning of CDS alerts and how to view drug information.

Participants were instructed as follows: (1) Approach tasks as if they were treating a real patient, exercising all due care; (2) Should they detect any prescribing errors, these should be addressed by not prescribing that medicine; (3) If the error involved an adverse drug interaction between two medicines, only one should be omitted; (4) If there was a discrepancy between CDS and the drug information they should rely on the drug information.

The task was presented as an evaluation of an e-prescribing system under development and participants were told that “Initial testing has shown that alerts are highly accurate, but occasionally have been incorrect. Therefore, you should always double check with the inbuilt drug information reference.” No information was provided on what types of errors the system would check and alert. Once all scenarios were completed, participants completed a post-experiment questionnaire and were then debriefed.

### Outcome measures

The present study was designed to test and analyze the following decision errors:

1. Omission errors: Where the participant failed to detect a genuine prescribing error. If the error was corrected by the participant, for example, by reducing a harmful dose to a safe level, it was not scored as an omission error.
2. Commission errors: Where the participant did not prescribe a safe medicine because of a false positive alert.

Prescribing errors were classified according to the definitions of prescribing error categories provided by Westbrook et al. [28]. The potential severity of prescribing errors was assessed by a clinical pharmacist (LGP) using the severity error classification scheme described in Dornan et al. [24].

### Statistical analyses

The presence of AB was tested using McNemar’s test [29] comparing errors between scenarios with incorrect CDS and scenarios with no CDS (control). It was estimated that 120 participants would be required to detect a 25% or greater difference (two-tailed) in errors between the control and incorrect CDS scenarios with 80% power and *p* < 0.05 [30]. With five hypotheses tested, a Bonferroni correction was applied to control for the increased risk of making a Type I error when testing multiple hypotheses [31]. With the desired alpha of 0.05, the corrected alpha against which all significance probabilities were evaluated became 0.01. Significance probabilities are only reported for comparisons between individual conditions, but not for aggregate figures by quality of CDS, which include multiple observations from each participant. Scenarios in which participants did not experience an interruption were excluded from the interruption analysis (*n* = 36 with no CDS, *n* = 22 with correct CDS, and *n* = 4 with incorrect CDS).

## Results

The participants’ average age was 24 years, and 46.7% were female. The majority rated their knowledge of medicines as fair (55.8%, *n* = 67) and only 5.8% (*n* = 7) reported previous training in e-prescribing systems. One participant completed the experiment twice (on two separate occasions), and the data from their second attempt was excluded.

In total, participants prescribed 4,065 medicines and made 1,049 prescribing errors (Table 1). This included 440 necessary medicines that were not prescribed. Of the total errors, 735 (70%) errors stemmed from opportunities the experiment provided for participants to make omission or commission errors. The remaining 314 (30%) where user-originated errors, independent of the experiment design and the majority of these were transcription errors. All participants made one or more prescribing errors. Compared to the control, correct CDS decreased prescribing errors by 58.8%, while incorrect CDS increased errors by 86.6%.

**Table 1:** Prescribing errors

		Control		Quality of Decision Support					
		No CDS		Correct CDS		Incorrect CDS		Total	
		n	%	n	%	n	%	n	%
Omission errors									
	Wrong drug	57	35.8	7	25.0	116	41.9	180	38.8
	Wrong dose	55	34.6	9	32.1	72	26.0	136	29.3
	Wrong frequency	0	0.0	0	0.0	0	0.0	0	0.0
	Drug-drug interaction	28	17.6	7	25.0	60	21.7	95	20.5
	Wrong route	0	0.0	0	0.0	0	0.0	0	0.0
	Wrong formulation	0	0.0	0	0.0	0	0.0	0	0.0
	Duplicated drug therapy	19	11.9	5	17.9	29	10.5	53	11.4
	Not indicated	0	0.0	0	0.0	0	0.0	0	0.0
	Not Prescribed	0	0.0	0	0.0	0	0.0	0	0.0
	Total omission errors	159		28		277		464	
Commission errors									
	Not prescribed	24	100.0	18	100.0	229	100.0	271	100.0
	Total commission errors	24		18		229		271	
User originated errors									
	Wrong drug	8	5.8	10	11.6	9	9.9	27	8.6
	Wrong dose	43	31.4	29	33.7	22	24.2	94	29.9
	Wrong frequency	10	7.3	6	7.0	4	4.4	20	6.4
	Drug-drug interaction	0	0.0	0	0.0	0	0.0	0	0.0
	Wrong route	0	0.0	0	0.0	1	1.1	1	0.3
	Wrong formulation	1	0.7	0	0.0	0	0.0	1	0.3
	Duplicated drug therapy	0	0.0	0	0.0	1	1.1	1	0.3
	Not indicated	0	0.0	1	1.2	0	0.0	1	0.3
	Not prescribed	75	54.7	40	46.5	54	59.3	169	53.8
	Total user originated errors	137		86		91		314	
Total errors									
	Wrong drug	65	20.3	17	12.9	125	20.9	207	19.7
	Wrong dose	98	30.6	38	28.8	94	15.7	230	21.9
	Wrong frequency	10	3.1	6	4.5	4	0.7	20	1.9
	Drug-drug interaction	28	8.8	7	5.3	60	10.1	95	9.1
	Wrong route	0	0.0	0	0.0	1	0.2	1	0.1
	Wrong formulation	1	0.3	0	0.0	0	0.0	1	0.1
	Duplicated drug therapy	19	5.9	5	3.8	30	5.0	54	5.1
	Not indicated	0	0.0	1	0.8	0	0.0	1	0.1
	Not Prescribed	99	30.9	58	43.9	283	47.4	440	41.9
	Total errors	320		132		597		1049	

Although participants were instructed to omit medicines they believed to contain prescribing errors, there were 43 instances where it appeared participants had substituted medicines not included in the scenario in an attempt to correct errors. Of these, 36 substitutions were replacing medicines associated with genuine prescribing errors, six were in response to false positive alerts, and one substitution was for a medicine not associated with any experimental manipulation.

### Correct CDS decreased prescribing errors

There were 40.8% fewer omission errors in scenarios with correct CDS compared to scenarios with no CDS (Table 2). This was significant across all levels of task complexity, with 38.3% fewer errors in low complexity (*p* < .0001, *n* = 120), 46.6% fewer errors in low + interrupt (*p* < .0001, *n* = 70), and 39.2% fewer errors in high complexity scenarios (*p* < .0001, *n* = 120).

**Table 2:** Number of participants making omission and commission errors

Scenario complexity	Quality of Decision Support											
	Control (No CDS)				Correct CDS				Incorrect CDS			
	Omission		Commission		Omission		Commission		Omission		Commission	
	No alert		No alert		True positive alert		True negative alert		False negative alert		False positive alert	
	(*n* = 120)		(*n* = 120)		(*n* = 120)		(*n* = 120)		(*n* = 120)		(*n* = 120)	
	n	%	n	%	n	%	n	%	n	%	n	%
Low	55	45.8	4	3.3	9	7.5	5	4.2	95	79.2	83	69.2
Low + Interrupt^a^	46	54.8	5	6	8	8.2	1	1	92	79.3	69	59.5
High	58	48.3	15	12.5	11	9.2	12	10	90	75	77	64.2
Total	159	49.1	24	7.4	28	8.3	18	5.3	277	77.8	229	64.3

^a^Number of participants in low + interrupt conditions: Control (*n* = 84), Correct DS (*n* = 98), and Incorrect DS (*n* = 116)

However, correct CDS did not significantly alter commission errors (Table 2). This was the case for low complexity (*p* = 1.0, *n* = 120), low + interrupt (*p* = .219, *n* = 65) and high complexity scenarios (*p* = .678, *n* = 120). Participants also made omission errors by overriding correct CDS alerts in 8.3% of scenarios and commission errors by not prescribing the safe, comparator, medicines in 5.3% of scenarios.

### Incorrect CDS increased prescribing errors

Participants missed 28.7% more genuine prescribing errors (omission errors) when assisted by incorrect CDS compared to no CDS (Table 2). These differences were statistically significant across all levels of complexity, with 33.3% more errors in low complexity (*p* < .0001, *n* = 120), 24.5% more errors in low + interrupt (*p* = .009, *n* = 82) and 26.7% more errors in high complexity scenarios (*p* < .0001, *n* = 120).

Overall participants made 56.9% more commission errors (did not prescribe safe medicines) when they received false positive alerts from incorrect CDS compared to when they received no CDS (Table 2). These differences were statistically significant across all levels of complexity, with participants in scenarios receiving false positive alerts making 65.8% more errors in low complexity (*p* < .0001, *n* = 120), 53.5% more errors in low + interrupt (*p* < .0001, *n* = 82) and 51.7% more errors in high complexity scenarios (*p* < .0001, *n* = 120).

### Interruptions to prescribing and scenario complexity did not impact automation bias

Interruptions did not affect omission or commission errors, or errors in the control scenarios. In interrupted scenarios with incorrect CDS there were 0.1% more omission errors (*p* = 1.0 *n* = 116) and 9.7% fewer commission errors (*p* = .08, *n* = 116). In interrupted control scenarios there were 8.9% more omission errors (*p* = .2, *n* = 84) and 2.6% more commission errors (*p* = .22, *n* = 84). All of these were non-significant.

Scenario complexity did not affect omission or commission errors, or errors in the control scenarios. In high complexity scenarios with incorrect CDS there were 4.2% fewer omission errors (*p* = .46, *n* = 120) and 5.0% fewer commission errors (*p* = .35, *n* = 120). In high complexity control scenarios there were 2.5% more omission errors (*p* = .75, *n* = 120) and 9.2% more commission errors (*p* = .007, *n* = 120). The only significant difference was between low and high complexity control scenarios.

### More omission than commission errors

Overall participants made 13.5% more omission than commission errors when provided with incorrect CDS, however, this was only significant in the low complexity + interrupt condition, all others were non-significant. There were 10% more omission errors in low complexity (*p* = .065, *n* = 120), 19.8% more in low + interrupt (*p* = .001, *n* = 116) and 10.8% more in high complexity scenarios (*p* = .079, *n* = 120).

## Discussion

### Main findings

This is the first study to find evidence of automation bias in the presence of e-prescribing CDS alerts. We found that when CDS was correct it reduced overall prescribing errors by 58.8%. This is consistent with prior literature showing that e-prescribing CDS can reduce prescribing errors [3–5]. However, when CDS was incorrect it increased errors by 86.6%. This increase was due to AB, that is, the ability of incorrect CDS to adversely influence participant prescribing decisions.

We found evidence of participants making omission errors, by failing to detect 28.7% more prescribing errors when CDS failed to provide alerts, compared to a control condition with no CDS. This finding was significant across all levels of task complexity and is potentially serious as the missed prescribing errors were classified as being of significant to potentially lethal severity, with most classified as serious severity.

Likewise, participants made commission errors, acting on clinically incorrect, false positive alerts, by not prescribing 56.9% more necessary medicines compared to the control condition. This was significant across all levels of task complexity.

These findings are consistent with and add to the research on automation bias in healthcare. Finding evidence of omission errors in the computer-aided detection of cancers in screening mammography [15, 16] and commission errors in the computerized interpretation of EKGs, [17] answering clinical questions assisted by CDS, [18] and deciding what to prescribe for clinical scenarios [19].

Interestingly, while participants were found to over-rely on automation, there was evidence of disagreement with the CDS provided to them. Participants’ overrode correct alerts and in doing so made prescribing errors which CDS was warning them to avoid. They also did not prescribe medicines which did not contain errors and for which there were no alerts. Reasons provided for overriding correct CDS alerts commonly referred to the condition for which the medicine was intended to treat (e.g. “VTE risk and pain management”, “vomiting”) or indicated that the medicine was regularly taken by the patient (e.g. “patient usual dose”). Participants commonly cited the lack of a true contraindication as the reason for overriding incorrect CDS alerts with many referring to the drug information. For example, “There is not any interaction listed on the drug information”. However, regular patient medicines and the condition treated were also mentioned as reasons for overriding incorrect CDS alerts. This suggests that not only did participants have trouble determining when CDS was wrong, but some also had trouble recognizing when it was right and that the alerts, or lack thereof, were beneficial and should be heeded.

#### Interruptions and task complexity did not impact automation bias

Interruptions did not affect the rate of AB errors nor did it affect errors rates in the control condition. However, interruptions are a complex phenomenon where multiple variables, including the characteristics of primary tasks, an individual’s cognitive state, the interruptions themselves, and the environment, may influence impact on clinical tasks and errors [22]. Despite clear evidence that interruptions can disrupt clinical tasks, their effects are complex, and may not always be detected [32].

Any impact of interruptions on prescribing errors was not detected in our experiment, replicating earlier results [33]. In our experiment, upon task resumption participants had ample time to recall their next action and the task environment provided cues to aid task resumption, for example, partly completed orders were visible on screen. One possible reason for not detecting an effect of interruptions was thus that disruption were minimized by these cues within the user interface [34]. This is consistent with observations from other studies of interruptions to computer-based tasks where participants were aided by the screen environment and were able to resume an interrupted task [35, 36]. Performance under cognitive load from more demanding competing tasks in a clinical environment may have resulted in a different outcome.

Contrary to expectations, the task complexity manipulation also had no effect on AB errors. This is in stark contrast to the findings of Bailey and Scerbo [25] who found performance on a system monitoring task deteriorated with increased task complexity, which they defined in terms of the cognitive demands placed on the participant. Monitoring tasks required the identification of critical deviations outside the normal operating range. Less complex tasks had participants monitor analogue gauges with marked critical regions. More complex tasks involved monitoring a display showing raw numbers where the subject had to remember the critical values for four different types of parameters.

Had the complexity manipulation altered the difficulty of prescribing task we would have expected to see a higher error rate in the high complexity control conditions. However, the observed difference was small and non-significant. This is in contrast to findings of Goddard et al. [19]. who found a significant effect for task difficulty, as classified by a panel of practitioners, on decision accuracy without CDS between medium and difficult scenarios. However, they found that task difficulty had no effect on commission errors.

The high error rate for both high and low levels of complexity in control conditions, with participants missing nearly half of all prescribing errors, seems to indicate that the difference in complexity between the two conditions may not have been large enough for differences in error rates to emerge.

### Implications

When clinical decision support is right, it can reduce prescribing errors by providing an important opportunity to detect and recover from prescribing errors. However, the finding of automation bias suggests that this additional layer of defence weakens or, at worst, becomes a replacement for the clinician’s own efforts in error detection with error detection delegated to CDS, without adequate oversight.

An intuitive solution to the problem of AB is to produce CDS systems that are less prone to error. While this may reduce the overall error rate, highly accurate automation is known to increase the rate of AB [25]. In other words, when automation does fail, the clinician will be even less able to detect it.

A key problem is that users seem to have difficulty in determining when CDS should and should not be relied on. Indeed, human factors research reports an inverse relationship between measures of verification, such as viewing drug references, and AB commission errors [10, 11]. So far, interventions to counter AB have had little success [37–39]. These include a number specifically targeted at verification, such as exposure to automation failures; [10] training about AB; and providing prompts to verify [40]. Compounding this problem further are findings of a *looking-but-not-seeing* effect or *inattentional blindness* where participants have made AB errors despite accessing sufficient information to assess that automation was incorrect [12, 13].

Verification, the means by which a user can determine whether the CDS they receive is correct, is key to the mitigation of AB. However, the lack of successful interventions indicates that more research is needed on how to best assist users with this crucial task.

This study has established that there is a risk of automation bias in electronic prescribing with senior medical students, who will soon be entering clinical practice as junior doctors. In doing this, we have also demonstrated a methodology for detecting AB in e-prescribing. The true rates and effects of AB in working clinical settings will require further studies and indeed is likely to vary by clinicians’ experience and familiarity with medications, clinical setting, patient complexity, and the particular decision support system used. All this is future work. Likewise, the lack of an effect of task complexity, even in control conditions, was surprising and something future studies will need to address. This might be achieved by varying clinician experience with prescribing and e-prescribing systems. Complexity could also incorporate familiarity with medicines, varying between simple, commonly-used to complex, rarely-prescribed regimes.

Clinicians need to be mindful that CDS can and does fail [6]. Ideally, clinicians should make every effort to detect prescribing errors, allowing CDS to function as an independent check for errors rather than relying on it as a replacement of their own error detection efforts.

### Limitations

Several limitations arise from the design of this study. While participants were instructed to approach the task as if they were treating a real patient, exercising all due care, the prescribing task was simulated, and prescribing errors were without consequence.

Also as an experiment, we cannot make any inferences about the true effect size or rate of AB in clinical settings as this will vary with, the user, the tasks being performed and the accuracy of the decision support provided. Likewise, the nature and incidence of the provided opportunities for prescribing errors may not be representative of those encountered in clinical practice.

The lack of a difference in prescribing errors between the low and high complexity control scenarios limited our ability to assess the impact of task complexity on AB.

Finally, the use of medical students with little experience in both prescribing medicines and using e-prescribing systems provides an indication of how CDS will impact new clinicians entering practice but limits generalisability for experienced prescribers or clinicians with e-prescribing experience.

## Conclusion

This study set out to test for the presence of automation bias in e-prescribing, a clinical decision support system commonly encountered by clinicians. We found evidence of omission errors, where participants failed to detect prescribing errors that were not alerted by CDS and commission errors, where participants acted on clinically incorrect alerts. Contrary to expectations, task complexity and interruptions had no impact on AB errors. However, when prescribing errors were correctly alerted, there was a dramatic reduction in the number of prescribing errors, demonstrating the benefits of CDS.

The challenge is to maximize the benefits of CDS while minimizing the risk of over-reliance. The key to this is enabling clinicians to determine when the CDS provided to them is correct, which is achieved through verification. Unfortunately, interventions tested to date, including those which focus on verification have produced little success. More research is needed on how to best assist clinicians with the task of verifying automation.

## Additional file

Additional file 1: Appendix A Overview of prescribing scenarios and Appendix B Example of an interruption task. (PDF 113 kb)

## Abbreviations

AB: Automation Bias
CDS: Clinical Decision Support
EKG: Electrocardiogram
e-prescribing: Electronic Prescribing
