# Supplementary material for: Automation bias in electronic prescribing
Source: BMC Med Inform Decis Mak. 2017 Mar 16;17:28. doi: 10.1186/s12911-017-0425-5 (PMC5356416; doi:10.1186/s12911-017-0425-5)
Supplement: Additional file 1: — Appendix A Overview of prescribing scenarios and Appendix B Example of an interruption task. (PDF 113 kb) [file 12911_2017_425_MOESM1_ESM.pdf]

Appendix A: Overview of prescribing scenarios

Scenario	Error Type	Medication	Alert displayed to participants	Comment	Severity
**A Low complexity**	Prescribing Error	Digoxin 250 microgram tablet, 2 tablets, PO, three times a day.	WARNING: High Dose<br>The entered dose is higher than the recommended maintenance dose range.	The elderly patient has atrial fibrillation which was controlled with Digoxin prior to admission.<br>The dose requested by the scenario is a loading dose. The maintenance dose for an elderly patient is 62.5 to 125 micrograms once daily.	Serious
	False Positive	Lisinopril 5mg tablet, 1 tablet, PO, once daily.	WARNING: Medicine Contraindicated<br>This medication is contraindicated for a condition (Pulmonary Oedema) contained in the patient record.	Lisinopril is not contraindicated in patients with pulmonary oedema.	
**B Low complexity**	Prescribing Error	Spironolactone 25mg tablet, 1 tablet, PO, once daily.	WARNING: Medicine Contraindicated<br>This medicine is contraindicated for a condition (Hyperkalaemia) contained in the patient record.	Patient has hyperkalaemia for which Spironolactone is contraindicated.	Serious
	False Positive	Augmentin Duo Forte (amoxycillin 875 mg + clavulanic acid 125 mg) tablet, 1 tablet, PO, twice daily.	WARNING: Medicine Contraindicated<br>This medication is contraindicated for a condition (Systolic Heart Failure) contained in the patient record.	Augmentin Duo Forte is not contraindicated in patients with heart failure.	
**C Low complexity**	Prescribing Error	Warfarin Sodium 2 mg tablet, 1 tablet, PO, once daily.<br>and<br>Ibuprofen 400 mg tablet, 1 tablet, PO, three times daily.	WARNING: Adverse Drug Interaction<br>This medication has a listed adverse interaction with another already prescribed medication.<br>There is an adverse drug interaction for Warfarin and Ibuprofen. This combination should be avoided.	Non-steroidal anti-inflammatory drugs (such as Ibuprofen) increase the risk of gastrointestinal bleeding in patients taking Warfarin. The combination should be avoided, especially as better analgesic options are available.	Significant
	False Positive	Atorvastatin 10 mg tablet, 1 tablet, PO, once daily.	WARNING: Medicine Contraindicated<br>This medication is contraindicated for a condition (Venous Thromboembolism) contained in the patient record.	Atorvastatin is not contraindicated in patients with venous thromboembolism.	
**D Low complexity**	Prescribing Error	Aspirin 300 mg tablet: effervescent, 3 tablets, PO, every 6 hours.	WARNING: Medicine Contraindicated<br>This medication is contraindicated for a condition (Peptic Ulcer Disease) contained in the patient record.	Patient has peptic ulcer disease with a history of bleeds for which aspirin increases the risk of gastrointestinal ulceration. There are better analgesic options.	Significant
	False Positive	Pantoprazole 40 mg tablet: enteric, 1 tablet, PO, once daily.	WARNING: Medicine Contraindicated<br>This medication is contraindicated for a condition (Severe Vomiting) contained in the patient record.	Pantoprazole is not contraindicated in patients with severe vomiting.	
**E Low complexity**	Prescribing Error	Loperamide Hydrochloride 2 mg capsule, 1 capsule, PO, PRN, every four hours, maximum 8 capsules per day.	WARNING: Medicine Contraindicated<br>This medication is contraindicated for a condition (Ulcerative Colitis) contained in the patient record.	Loperamide is contraindicated in patients with ulcerative colitis which poses a risk of toxic megacolon.	Serious
	False Positive	Mesalazine 500 mg tablet: enteric, 1 tablet, PO, three times daily.<br>and<br>Prednisolone 25 mg tablet, 1 tablet, PO, once daily.	WARNING: Adverse Drug Interaction<br>This medication has a listed adverse interaction with another already prescribed medication.<br>There is an adverse drug interaction for Mesalazine and Prednisolone. This combination should be avoided.	There is no documented adverse drug interaction for Mesalazine and Prednisolone.	

Scenario	Error Type	Medication	Alert displayed to participants	Comment	Severity
**F**<br>**Low complexity**	Prescribing Error	Phenelzine 15 mg tablet, 1 tablet, PO, three times daily.<br>and<br>Tramadol Hydrochloride 50mg capsules, 2 capsules, PO, PRN, every six hours, maximum 8 capsules per day.	WARNING: Adverse Drug Interaction<br>This medication has a listed adverse interaction with another already prescribed medication.<br>There is an adverse drug interaction for Phenelzine and Tramadol hydrochloride. This combination should be avoided.	The combination of phenelzine and tramadol are contraindicated due to the possibility of causing serotonin toxicity.	Serious
	False Positive	Ramipril 5 mg tablet, 1 tablet, PO, once daily.	WARNING: Adverse Drug Reaction<br>This patient has an Allergy or Adverse Drug Reaction recorded for this medication.	The patient is allergic to Sulfonamide. However Ramipril is not contraindicated for this allergy.	
**G**<br>**High complexity**	Prescribing Error	Paracetamol 500 mg tablet, 2 tablets, PO, four times a day.<br>and<br>Panadeine Forte (Codeine Phosphate with Paracetamol Tablet 30 mg-500 mg) tablet, 2 tablets, PO, PRN, every four hours, maximum 8 tablets per day.	WARNING: High Dose / Duplicate Substance<br>Both Paracetamol and Panadeine Forte (Codeine Phosphate 30mg with Paracetamol 500mg) contain the ingredient Paracetamol. The total Paracetamol entered is higher than the recommended dose range.	Prescribed together these two prescriptions provide for a combined maximum possible dose of 8 grams of paracetamol per day, double the maximum daily dose of 4 grams.	Significant
	False Positive	Ciprofloxacin 250 mg tablet, 1 tablet, PO, twice daily.	WARNING: Adverse Drug Reaction<br>This patient has an Allergy or Adverse Drug Reaction recorded for this medication.	The patient is allergic to penicillin. Ciprofloxacin is an antibiotic, however, it is not contraindicated for allergy to penicillin.	
**H**<br>**High complexity**	Prescribing Error	Methotrexate 2.5 mg tablets, 3 tablets, PO, once daily.	WARNING: High Dose<br>The entered dose is higher than the recommended maintenance dose range	Patient has net onset rheumatoid arthritis. For treatment of rheumatoid arthritis, the loading dose of methotrexate is 7.5mg once weekly.	Potentially lethal
	False Positive	Paracetamol 500 mg tablet, 2 tablets, PO, PRN, every four hours, maximum 8 tablets per day.	WARNING: Medicine Contraindicated<br>This medication is contraindicated for a condition (Peptic Ulcer Disease) contained in the patient record.	Patient has newly diagnosed peptic ulcer disease, however, it is not a contraindication for paracetamol.	
**I**<br>**High complexity**	Prescribing Error	Metoclopramide Hydrochloride 10 mg tablet, 1 tablet, PO, PRN, three times daily, maximum 3 tablets per day.	WARNING: Medicine Contraindicated<br>This medication is contraindicated for a condition (Parkinson's Disease) contained in the patient record.	Patient has a history of Parkinson's disease for which Metoclopramide is contraindicated as symptoms may worsen. The drug reference provides an alternative medicine as being preferred.	Serious
	False Positive	Entacapone 200 mg tablet, 1 tablet, PO, three times daily.<br>and<br>Rosuvastatin 20 mg tablet, 1 tablet, PO, once daily.	WARNING: Adverse Drug Interaction<br>This medication has a listed adverse interaction with another already prescribed medication.<br>There is an adverse drug interaction for Entacapone and Rosuvastatin. This combination should be avoided.	There is no documented adverse drug interaction for Entacapone and Rosuvastatin.	

Appendix B: Example of an interruption task

Harold O'Brien is about to commence Vancomycin for meningitis. Please refer to the test Creatinine Clearance in the test results provided and select the appropriate dose and frequency of administration using the dosing tables below.

**Clinical Chemistry**		
Sodium (mmol/L)	**140**	135-145
Potassium (mmol/L)	**4.5**	3.5-5.0
Chloride (mmol/L)	**106**	95-107
Bicarbonate (mmol/L)	**28**	24-32
Urea (mmol/L)	**9.2**	3.0-8.0
Creatinine (mmol/L)	**141**	60-110
Creatinine Clearance (mL/min)	**64**	97-137

**Creatinine clearance (mL/min)**	**Starting maintenance dosage**
more than 90	1.5 g
90 or less	1 g

**Creatinine clearance (mL/min)**	**Frequency**
more than 60	12-hourly
20 to less than 60	24-hourly
less than 20	48-hourly

Please review the information above. What *dose* of Vancomycin should be given and how *frequently* should it be administered?

(a) 1.5g, 12-hourly

(b) 1.5g, 24-hourly

(c) 1.5g, 48-hourly

(d) 1g, 12-hourly

(e) 1g, 24-hourly

(f) 1g, 48-hourly
